# Improved XLNet modeling for Chinese named entity recognition of edible fungus

**DOI:** 10.3389/fpls.2024.1368847

**Published:** 2024-06-25

**Authors:** Helong Yu, Chenxi Wang, Mingxuan Xue

**Affiliations:** College of Information Technology, Jilin Agricultural University, Changchun, China

**Keywords:** named entity recognition, edible fungus, XLNet, Chinese text, CRF

## Abstract

**Introduction:**

The diversity of edible fungus species and the extent of mycological knowledge pose significant challenges to the research, cultivation, and popularization of edible fungus. To tackle this challenge, there is an urgent need for a rapid and accurate method of acquiring relevant information. The emergence of question and answer (Q&A) systems has the potential to solve this problem. Named entity recognition (NER) provides the basis for building an intelligent Q&A system for edible fungus. In the field of edible fungus, there is a lack of a publicly available Chinese corpus suitable for use in NER, and conventional methods struggle to capture long-distance dependencies in the NER process.

**Methods:**

This paper describes the establishment of a Chinese corpus in the field of edible fungus and introduces an NER method for edible fungus information based on XLNet and conditional random fields (CRFs). Our approach combines an iterated dilated convolutional neural network (IDCNN) with a CRF. First, leveraging the XLNet model as the foundation, an IDCNN layer is introduced. This layer addresses the limited capacity to capture features across utterances by extending the receptive field of the convolutional kernel. The output of the IDCNN layer is input to the CRF layer, which mitigates any labeling logic errors, resulting in the globally optimal labels for the NER task relating to edible fungus.

**Results:**

Experimental results show that the precision achieved by the proposed model reaches 0.971, with a recall of 0.986 and an F1-score of 0.979.

**Discussion:**

The proposed model outperforms existing approaches in terms of these evaluation metrics, effectively recognizing entities related to edible fungus information and offering methodological support for the construction of knowledge graphs.

## 1 Introduction

Edible fungus is globally recognized as 21st-century health food. It serves as an important raw material for biopharmaceuticals, functional foods, washing products, and cosmetics, and is widely used for clothing bags and green buildings (Amobonye et al., 2023). China has abundant edible fungus resources, and was the first country to recognize, harvest, consume, and cultivate edible fungus. Since 2012, edible fungus (i.e., vegetable fungus) has emerged as the fifth-largest category among agricultural products, following food, oil, fruits, and vegetables (Li and Xu, 2022). The large number of edible fungus species and the vast amount of mycological knowledge pose great challenges to the research, cultivation, and dissemination of knowledge about edible fungus. In addressing this challenge, there is an urgent need for a method that can rapidly and accurately acquire information on edible fungus. The emergence of question and answer (Q&A) systems presents an opportunity to overcome this issue. Named entity recognition (NER) is now widely used in the military (Wang et al., 2018; Lu et al., 2020; Baigang and Yi, 2023; Li et al., 2023), entertainment and culture (Molina-Villegas et al., 2021; Zhuang et al., 2021; Fu et al., 2022; Huang et al., 2022), cybersecurity (Georgescu et al., 2019; Simran et al., 2020; Chen et al., 2021; Ma et al., 2021), and medicine (Ji et al., 2019; Li et al., 2020; Wang et al., 2020; Liu et al., 2022). However, the application of NER in the agricultural sector is still in the early stages of development (Wang et al., 2022; Yu et al., 2022a; Qian et al., 2023). Recognizing named entities related to edible fungus is fundamental in building an intelligent Q&A system dedicated to edible fungus. Such a system would play a significant role in advancing the research, cultivation, and dissemination of knowledge related to edible fungus.

Initial NER approaches typically employed dictionary- and rule-based methods. Cook et al. introduced a dictionary-based named entity tagger called TagIt, which was subsequently enhanced with a rule-based extension system to improve information extraction from the corpus (Cook et al., 2016). However, this method relies on a predefined dictionary for entity recognition, so an entity may not be recognized if the corresponding entry in the dictionary is incomplete, such as when a new word is absent. Additionally, the accuracy may be impacted if the dictionary has a limited number of entries, and the operational efficiency may be hindered is the dictionary contains an extensive number of words. Traditional machine learning-based NER methods include hidden Markov models (Yan et al., 2021), maximum entropy (Jain et al., 2022), support vector machines (Hamad and Abushaala, 2023), and conditional random fields (CRFs) (Liang et al., 2017).Traditional machine learning methods are superior to unlabeled NER, but are heavily dependent on manually selected features and require substantial amounts of training data to attain satisfactory performance.

The emergence of deep learning has enhanced the feature extraction capabilities during model training. Ji et al. and Li et al (Ji et al., 2018; Li et al., 2019). proposed models that integrate a language model with a CRF and a bidirectional long short-term memory (BiLSTM). They used these models to extract entities from Chinese electronic medical records (EMRs) and drug instructions, respectively. In 2018, the bidirectional encoder representations from transformers (BERT) was introduced as an alternative to Word2Vec. BERT achieved improved accuracy across 11 tasks in the field of natural language processing (Devlin et al., 2019). Dai et al. and Zhang et al. improved the semantic representation of words by leveraging the BERT pre-trained language model on the Chinese EMR dataset. They then combined the BiLSTM with a CRF layer, using the word vectors as inputs for training. Experimental results show that the BERT-BiLSTM-CRF model outperforms other baseline models (Zhang et al., 2019; Dai et al., 2019a). The implementation of the BERT-BiLSTM-CRF model to agricultural pests and illnesses by Liu et al. demonstrated its effectiveness for detecting entities related to citrus pest and disease (Liu et al., 2023). Although BiLSTM-CRF performs well in NER tasks, its limited ability to fully utilize GPU parallelism leads to suboptimal model performance.

Therefore, Strubell et al. proposed the iterated dilated convolutional neural network (IDCNN) (Strubell et al., 2017), which has the capability to capture longer contextual information than traditional CNNs and enables better parallelism than traditional LSTM units. Yu and Wei proposed an entity recognition method based on character embedding through IDCNN and CRF, and reported an F1-score of more than 94% in a test corpus based on military equipment (Yu and Wei, 2020). Cai et al. introduced a BERT-IDCNN-CRF model that enhanced the average accuracy, recall, and F1-score by 8.4%, 3.3%, and 6.2%, respectively, compared with a baseline model. This enhancement illustrates the model’s ability to identify medical terms in EMR (Cai et al., 2022). It can be seen that the introduction of the IDCNN layer to expand the sensory field of the convolutional kernel enhances the model’s ability to capture inter-utterance features. In addition, combining the CRF layer also ensures the logic and global optimality of the annotation.

Although BERT is acknowledged to have superior performance in NER tasks, it overlooks the dependency between masked positions. Thus, Yang et al. introduced XLNet to overcome this limitation (Yang et al., 2020). Abu-Salih and Alotaibi later constructed an XLNet-BiLSTM-CRF model, effectively capturing contextual information and sequential dependencies in customer engagement data. Their findings reveal that, in terms of precisely recognizing brand advocates and designating consumer advocacy entities, the XLNet-BiLSTM-CRF model performs better than more basic architectures (Abu-Salih and Salihah Alotaibi, 2023). So in this paper, we choose to combine XLNET with IDCNN and CRF.

To date, there have been no reports on the application of NER to edible fungus. Most of the literature in this field refers to the cultivation and application of NER in related fields (Carrasco and Preston, 2020; Kumar et al., 2021). Thus, there are two problems facing the NER task in the field of edible fungus:

(1) In the current stage of research on edible fungus, there is a lack of publicly available Chinese datasets suitable for the NER task.
(2) Certain entities within the text of edible fungus information may be positioned independently at the beginning or end of the text, posing a challenge in capturing features between statements and complicating the recognition process.

To tackle these issues, this paper describes the establishment of a Chinese corpus specifically designed for NER in the field of edible fungus. Furthermore, this paper introduces an IDCNN-CRF NER model called XIC. Using the XLNet model as the foundational framework, the IDCNN layer enhances the ability to capture features between utterances by expanding the receptive field of the convolutional kernel. The output of the IDCNN layer is input to the CRF layer to mitigate labeling logic errors and acquire the globally optimal labels.

The main contributions of this paper are as follows:

(1) The “Atlas of Chinese Large Fungus Resources,” authored with the participation of academician Li Yu and other experts in mycology, is transformed into a Chinese corpus in the field of edible fungus. This transformation involves data cleaning, formatting, and entity annotation specifically tailored for the task of NER.
(2) This paper introduces a method for NER in the field of edible fungus. The method addresses the gap in NER of edible fungus information by incorporating an IDCNN layer with the XLNet model, enhancing the capture of features between statements. Additionally, the introduction of a CRF layer improves the ability to obtain the globally optimal labeling sequence.
(3) In the domain of edible fungus, the model proposed in this paper demonstrates excellent performance in the task of NER and is applicable to related NER tasks.

## 2 Materials and methods

### 2.1 Dataset collection and labeling

#### 2.1.1 Dataset acquisition and preprocessing

To overcome the absence of publicly available textual data in the public domain of edible fungus, this study took the “Atlas of Chinese Macrofungal Resources,” published in December 2015 and edited by academician Li Yu, Prof. Tuligul, and others, as the primary data source. This seminal work serves as the foundation for constructing a dataset tailored for entity recognition experiments in the field of edible fungus. The species included in the “Atlas of Chinese Macrofungal Resources” are exclusively derived from Chinese resources and illustrated with meticulously verified taxonomic information. This rigorous taxonomic verification process ensures a comprehensive and objective representation of the actual situation of Chinese macrofungal resources, making the dataset a reliable foundation for our study. The “Atlas of Chinese Macrofungal Resources” serves as a comprehensive introduction to Chinese macrofungal resources, documenting 1819 species (or varieties) in 509 genera of macrofungi known in China. Based on their morphological characteristics, these species are systematically categorized into 10 groups, each introduced in a corresponding chapter. These groups include 196 larger ascomycetes, 21 jelly fungi, 47 coral fungi, 637 polyporoid, hydnaceous, and thelephoroid fungi, 11 cantharelloid fungi, 653 agarics, 130 boletes, 75 gasteroid fungi, 16 larger pathogenic fungi on crops, and 33 larger myxomycetes.

The raw dataset was subjected to data preprocessing to remove items such as pictures and tables, which may hinder the extraction of correct entity information. The process of data preprocessing encompassed both data cleaning and data formatting. Data cleaning primarily involved substituting redundant space breaks with nulls and removing extraneous information from the raw dataset. Given the presence of excessively lengthy text in the original dataset, which could potentially impact the entity recognition accuracy, data formatting was employed to segment the original dataset into clauses based on punctuation periods.

#### 2.1.2 Entity type classification

Building on the identification of data sources, entity types were defined according to dataset characteristics. Specifically, this study relied on the record sheet of the mycorrhizal field collection found in the “Atlas of Chinese Macrofungal Resources.” The entity type of mycorrhiza was determined through thorough analysis of the book’s contents. All species described in the “Atlas of Chinese Macrofungal Resources” are complemented by color photographs illustrating their macro-morphology and/or habitat. The book offers concise textual descriptions of the macroscopic and microscopic diagnostic characters, ecological habits, economic significance (including edibility, medicinal uses, or toxicity), and geographical range in China for each species. Under the guidance of experts and considering the characteristics of the literature content, 31 entity types and their corresponding labels in the NER model were formulated, as detailed in **Table 1**.

**Table 1:** Entity type.

Labels	Entity type	Number	Labels	Entity type	Number
CCNF	Chinese common name of the fungus	301	ASH	Ascospores shape	171
CNF	Chinese name of the fungus	1819	SICD	Size of the cystic disc	94
ADF	Area of distribution of the fungus	1818	SHCD	Shape of the cystic disc	90
BSF	Basic substance of fungus	1839	CDC	Cystic disc color	21
FGE	Fungal growth environment	1472	SSI	Sub-entity size	413
FE	Fungal ecology	757	SSH	Sub-entity shape	131
TC	Thickness of cap	384	SGC	Substrate growth cycle	630
CSI	Cap size (of fungus)	1208	SCO	Sub-entity color	98
CSH	Cap shape (of fungus)	1112	BSI	Basidiospore size	1570
CC	Cap color (of fungus)	1154	BSH	Basidiospore shape	1520
FT	Flesh thickness	617	GT	Growing time	1493
TMF	Texture of mushroom flesh	336	EU	Economic use	641
FC	Flesh color	1329	SIMS	Size of mushroom stipe	990
SIC	The size of the ascus	162	SHMS	Shape of mushroom stipe	688
SHC	The shape of the ascus	119	CMS	Color of mushroom stipe	828
ASI	Ascospore size	175			

#### 2.1.3 Text annotation

Data annotation facilitates the recognition and learning of unprocessed text by machines. This process typically involves automatic annotation and manual annotation. Automatic annotation uses machines and algorithms to identify text content, whereas manual annotation requires human annotators to identify and label the text content. Manual labeling is more accurate, but lags far behind automatic labeling in terms of efficiency. Due to the unique characteristics of the original dataset, this study employed manual annotation for data labeling.

The Chinese corpus in the field of edible fungus underwent manual labeling based on the entity types specified in the previous section. In this study, approximately 370,000 words from the “Atlas of Chinese Macrofungal Resources” were textually annotated, resulting in the identification of 23,980 entities. Existing text annotation methods encompass the three-digit annotation method BIO, the four-digit sequence annotation method BEMS, and the five-digit annotation method BIOES, which was employed in this study. The labeling convention used in this study designated the word-initial position of each entity as B, the word-final position as E, the middle word position as I, and a single word as S. To enhance the model’s recognition of entities, we appended “-Entity Type” after BIOES, as illustrated in **Table 2**. As shown in **Table 2**, ‘黄无座盘菌’ was labeled as the Chinese name of the fungus (CNF), and “直径0.5~1.5mm” was labeled as the size of the cystic disk (SICD). According to the BIOES annotation method, ‘黄’ was labeled as B-CNF, ‘菌’ was labeled as E-CNF, ‘无’, ‘座’, ‘盘’ were labeled as I-CNF, and all other entities were labeled according to the BIOES annotation method.

**Table 2:** Annotation examples.

Character	Label	Character	Label	Character	Label	Character	Label
黄	B- CNF	直	B- SICD	m	I- SICD	脓	I- SHCD
无	I- CNF	径	I- SICD	m	E- SICD	疱	I-SHCD
座	I- CNF	0	I- SICD	,	O	状	E-SHCD
盘	I- CNF	.	I- SICD	近	B-SHCD	,	O
菌	E- CNF	5	I- SICD	圆	I- SHCD	无	O
:	O	~	I- SICD	球	I- SHCD	柄	O
子	O	1	I- SICD	形	I- SHCD	。	O
囊	O	.	I- SICD	至	I- SHCD		
盘	O	5	I- SICD	小	I- SHCD		

### 2.2 Method

**Figure 1:**
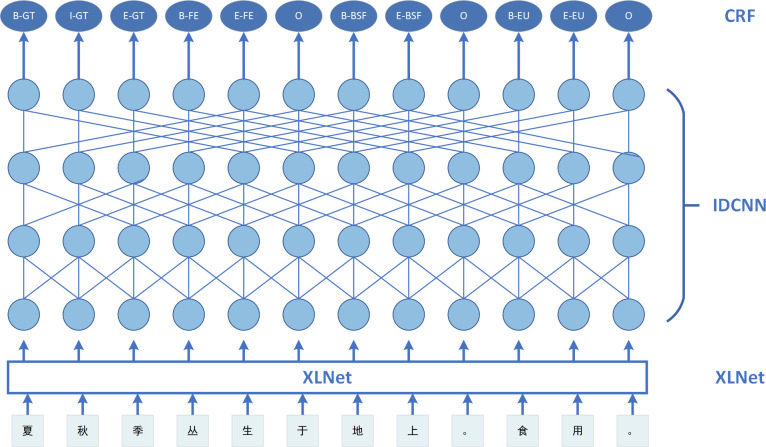
Model structure diagram.

This section outlines the structure of the XLNet-IDCNN-CRF model. The model structure is depicted in **Figure 1**. There are four layers, arranged from bottom to top as follows: the embedding layer, XLNet layer, IDCNN layer, and CRF layer. The work described in this paper is inspired by a prior study (Yu et al., 2022b) in which the characters generated by the last layer of the BERT model were embedded in a neural network. In our case, the XLNet model is employed instead of the BERT model, and the IDCNN is integrated into the XLNet model to enhance the capture of features between utterances. Furthermore, to obtain optimal labeling sequences, we used the CRF, as successfully applied in Chinese NER (Qiu et al., 2019) and Portuguese NER (Souza et al., 2019).

#### 2.2.1 XLNet model

The construction of the XLNet model (Yang et al., 2020) is grounded in the BERT architecture, with several improvements. XLNet deviates from the relatively fixed order of factorization in the previous autoregressive (AR) model, aiming to maximize the expected log-likelihood value. Simultaneously, XLNet reduces its dependence on residual data, effectively mitigating the data inconsistency issues that occur in BERT.

The primary essence of the XLNet model lies in the effective reconstruction of input text through permutations and combinations. This facilitates comprehensive analysis of contextual information for bidirectional prediction. The main purpose of introducing the permutation language model is to maximize the probability of likelihood, as shown in Equation 1.


(1) $$\begin{array}{c} MAX_{\theta }E_{Z-Q_{\theta }\;}\left[\sum_{t=1}^{T}lgp_{\theta }\left(x_{Z_{t}}|XZ_{<t}\right)\right] \end{array}$$

Among them, the calculation of P is shown in Equation 2.


(2) $$\begin{array}{c} P_{\theta }\left(x_{Z_{t}}|X_{Z_{<t}}\right)=\frac{exp\left(e{\left(x\right)}^{T}g^{\theta }\left(X_{Z_{<t}},\;Z_{t}\right)\right)}{\sum_{x^{\prime }}exp\left(e{\left(x^{\prime }\right)}^{T}g^{\theta }\left(X_{Z_{<t}},\;Z_{t}\right)\right)} \end{array}$$

During the pre-training stage, fine-tuning of the original input text cannot be achieved through organic arrangement and combination. Therefore, in the pre-training process, the order of the input text is often adjusted within the transformer. Uniquely, XLNet leverages an attention mask to accomplish this function. In the process of XLNet modeling, g^θ^(X_Z<t_, Z_t_) is realized through a two-stream self-attention mechanism.

To capture the semantic information of long-distance text, the XLNet model incorporates a recurrent mechanism and relative positional encoding, building upon the foundation of the Transformer-XL idea (Dai et al., 2019b). The loop mechanism extracts information from each layer of the previous sequence, serving as a memory cache for predicting the next segment. This enhances the training efficiency while effectively handling tasks involving long documents. Relative positional encoding is employed to prevent the loss of positional information, which provides a better characterization of word polysemy and improves text feature extraction. In this study, the XLNet model is employed to extract contextual features from a text dataset, facilitating the acquisition of bidirectional contextual information and the mining of long-range textual features. This approach proves beneficial for improving entity recognition in the field of edible fungus.

#### 2.2.2 Adding IDCNN to XLNet

**Figure 2:**
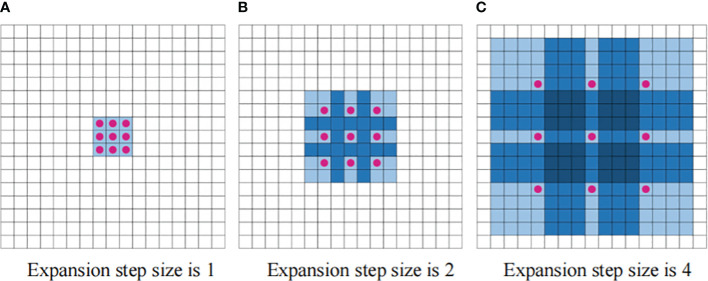
Dilated convolution diagram. **(A)** Expansion step size is 1 **(B)** Expansion step size is 2 **(C)** Expansion step size is 4.

Traditional CNNs typically require greater network depths or enlarged convolutional kernels to enhance the receptive field area. However, this approach results in the proliferation of network parameters, leading to computational difficulties and slow model convergence. Dilated convolution serves the primary purpose of expanding the perceptual field of view (Yu and Koltun, 2016). In classical CNNs, the convolution kernel slides over a continuous region. In contrast, dilated convolution introduces an expansion width. During the convolution operation, the data in the middle of the expansion width are skipped, while the size of the convolution kernel remains unchanged. This allows a convolution kernel of given size to capture a wider range of data in the input matrix, effectively increasing the receptive field of the convolution kernel. The process of convolution kernel expansion is illustrated in **Figure 2**.

The three convolutional blocks in **Figure 2** have expansion widths of 1, 2, and 4, resulting in corresponding receptive fields of sizes 3 × 3, 7 × 7, and 15 × 15, respectively. With the same convolutional kernel size, the receptive field increases exponentially with the expansion width. This rapid expansion enables the sensory domains to cover all input sequences.

Dilated convolution was originally applied in image processing. Strubell et al. (2017) later introduced dilated convolution into natural language processing and proposed the IDCNN model, which has proven to be effective. The model consists of four dilated CNN blocks, each with the same size and structure. Within each block, there are three dilated convolutional layers with dilation widths of 1, 1, and 2. Sentences are input into the XLNet model and features are extracted through the convolutional layer of the IDCNN. These features are then input to the CRF layer through the mapping layer.

The overall IDCNN-CRF model is similar to the classical BiLSTM-CRF model. Compared with recurrent neural networks, IDCNN addresses issues related to longer sequence information and achieves faster model training.

#### 2.2.3 Adding CRFs to XLNet-IDCNN

To prevent sequence labeling errors, such as the beginning of an entity being labeled with “I-CNF” or “E-CNF” instead of following the BIOES labeling logic, a CRF is introduced to process the feature recognition results of the IDCNN layer. By learning inter-entity dependencies through the CRF, the model decodes the optimal label sequences for sequence labeling.

For a given sequence $x=\left(x_{1},x_{2},x_{3},\cdots ,x_{n}\right)$ and the corresponding sequence of labels $y=\left(y_{1},y_{2},y_{3},\cdots ,y_{n}\right)$ , the IDCNN layer assigns scores for each label.The calculation formula is the Equation 3:


(3) $$\begin{array}{c} F_{i}=W_{s}O^{t}+b_{s} \end{array}$$

where $O^{t}$ represents the output corresponding to input data $x^{t}$ at moment *t* from the previous layer, and $W_{s}$ , $b_{s}$ are linear mapping parameters.

The CRF establishes a label transfer score, expressing the score from the input sequence of the text to the label sequence of the text as


(4) $$\begin{array}{c} s\left(x,y\right)=\sum_{i=1}^{m}\left(W_{y_{i-1,}y_{i}}+F_{i,y_{i}}\right) \end{array}$$

In Equation 4, W is the transformation matrix, $W_{i,j}$ represents the label transfer score, and $F{\;}_{i,yi}$ represents the score of the $y_{i}$ th label of the character. The matrix W = ( $W_{u,\;j}$ ). The maximum likelihood function for the training set $\left\{x_{i},y_{i}\right\}$ is as shown in the Equation 5.


(5) $$\begin{array}{c} L=\sum_{i=1}^{m}\log\left(P\left(y_{i}|x_{i}\right)\right)+\frac{\alpha }{2}\|\beta {\|}^{2} \end{array}$$

where α and β are regularization parameters and P represents the probability of the original sequence corresponding to the predicted sequence, as shown in the Equation 6:


(6) $$\begin{array}{c} P\left(y|x\right)=\frac{e^{s\left(x,y\right)}}{\sum_{y\in Y_{x}}e^{s\left(x,y\right)}} \end{array}$$

### 2.3 Experimental setup

In this study, the dataset was subjected to word splitting using the Jieba library. The configuration of the experimental environment for this paper is as follows: for hardware, we used an Intel(R) Xeon(R) Gold 5218R CPU at 2.10 GHz and an NVIDIA GeForce RTX 2080 Ti graphics card. For the operating system, we chose Windows 10 Professional and configured CUDA 12.2 to support GPU-accelerated computing. For the software configuration, we used Python 3.8 as the programming language and installed PyTorch 1.12.1 as the deep learning framework. In addition, to handle data and matrix operations, we installed the NumPy 1.23.5 library. Also, we used the Transformers 4.24.0 library, which is a Python library specialized for natural language tasks. The whole experimental environment provides strong support and guarantee for us to carry out deep learning and natural language processing tasks.

The labeled predictions were partitioned into training, validation, and test sets with an 8:1:1 ratio. The training set comprises 19,184 entities, and the validation and test sets each contain 2,398 entities.

### 2.4 Evaluation indicators

Three evaluation metrics were used to assess the proposed model-the precision P, recall R, and F1-score, as shown in the Equations 7–9. R represents the ratio of correctly labeled entities to the total number that should have been labeled, P is the ratio of correctly labeled entities to the total number labeled, and the F1-score is the harmonic average of the two. These metrics are calculated as


(7) $$\begin{array}{c} P=\frac{TP}{TP+FP} \end{array}$$

(8) $$\begin{array}{c} R=\frac{TP}{TP+FN} \end{array}$$

(9) $$\begin{array}{c} F1=\frac{2RP}{R+P} \end{array}$$

where TP (true positive) denotes a true example, signifying cases where both the predicted and actual results are entities related to edible fungus information, FP (false positive) represents a pseudo-positive example, indicating instances where the actual results are entities unrelated to edible fungus information, but the predicted results are entities related to edible fungus information, FN (false negative) denotes a pseudo-negative example, reflecting situations where the actual result is an entity related to edible fungus, but the predicted result is an entity unrelated to edible fungus information.

## 3 Results and discussion

### 3.1 Ablation experiments

To assess the effectiveness of the IDCNN-CRF model introduced in this paper, the results of ablation experiments are now presented.

#### 3.1.1 Performance comparison between XLNet and XLNet-IDCNN

Pre-trained feature vectors of edible fungus information were used as inputs to compare the recognition performance of XLNet and XLNet-IDCNN, as illustrated in **Table 3**. The P, R, and F1-scores of the XLNet-IDCNN model are 0.878, 0.961, and 0.917, respectively. Notably, P, R, and F1 exhibit improvements of 0.416, 0.166, and 0.333, respectively, in comparison with the XLNet model. These results indicate that the addition of the IDCNN layer to the XLNet model effectively enhances the recognition performance of the model.

**Table 3:** Adding IDCNN named entity recognition results.

Model	P	R	F1
XLNet	0.462	0.795	0.584
XLNet-IDCNN	0.878	0.961	0.917

**Figure 3:**
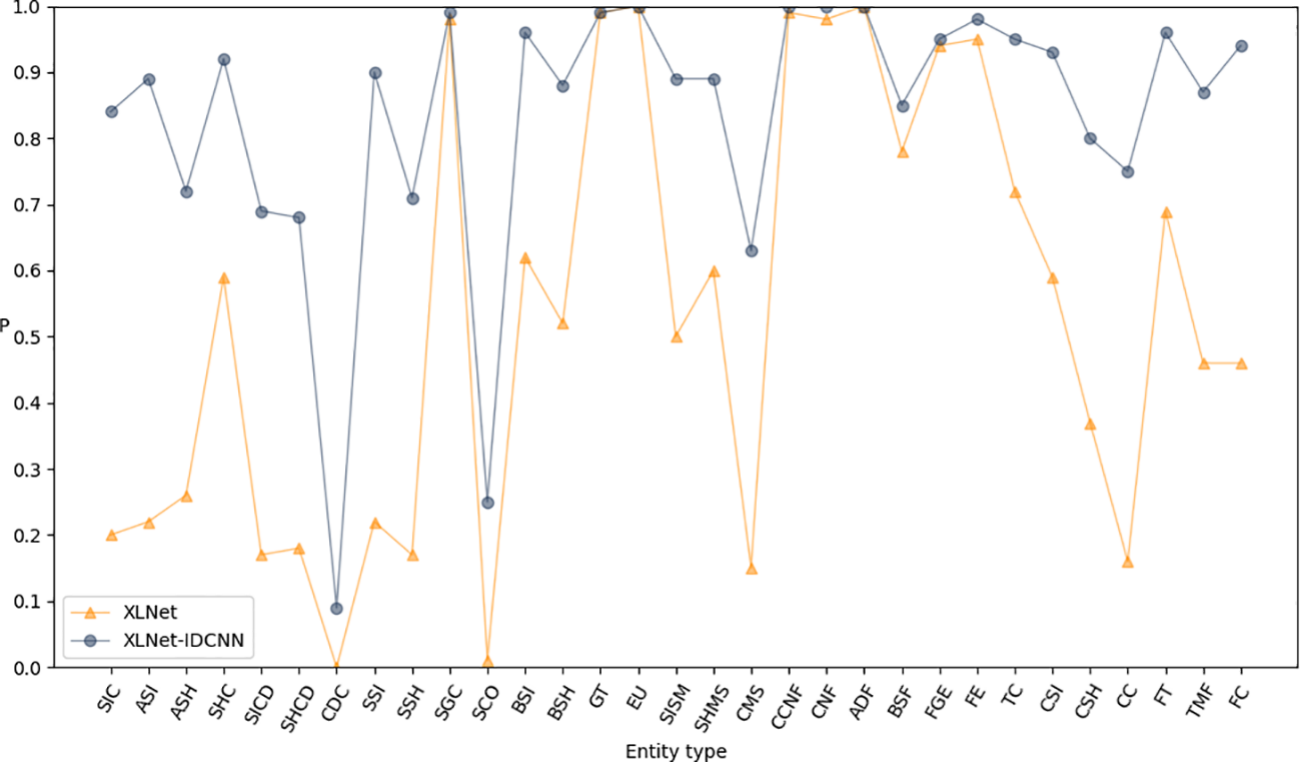
P-values of XLNet and XLNet-IDCNN on different entities.

**Figure 4:**
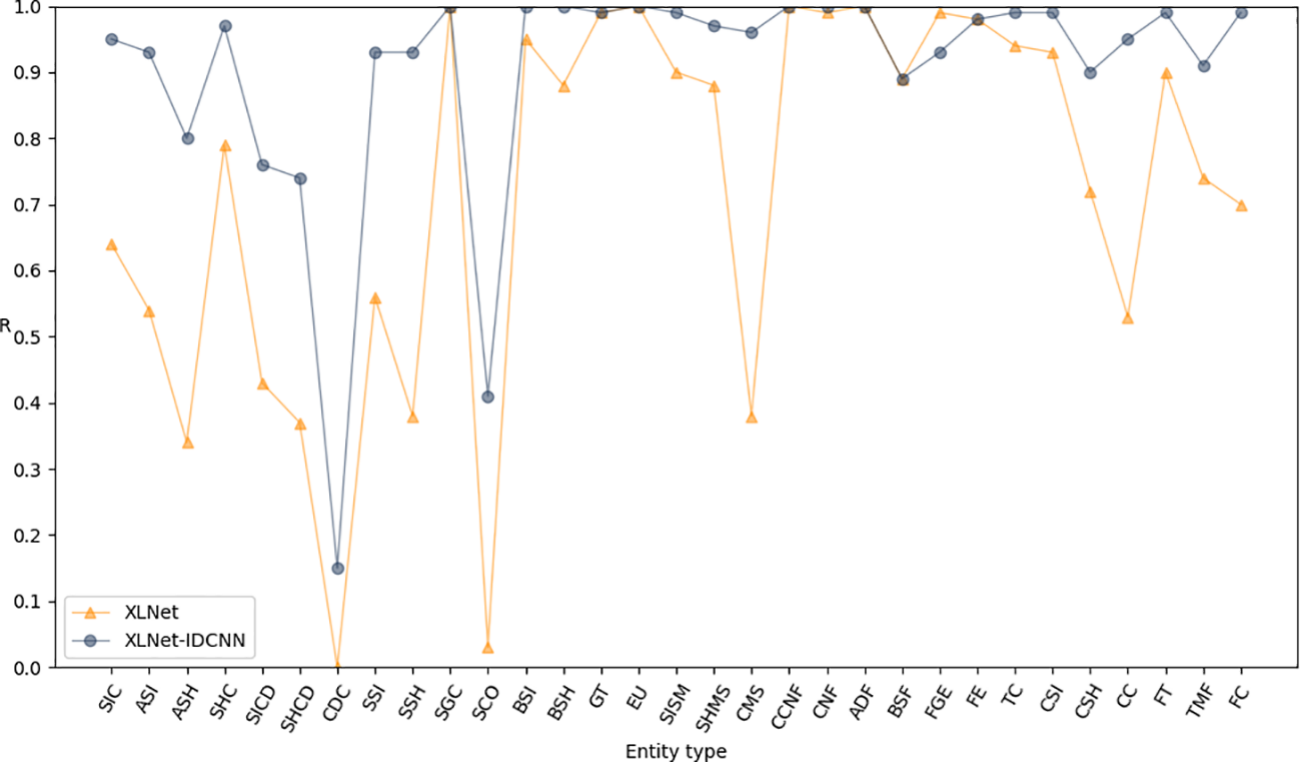
R-values of XLNet and XLNet-IDCNN on different entities.

**Figure 5:**
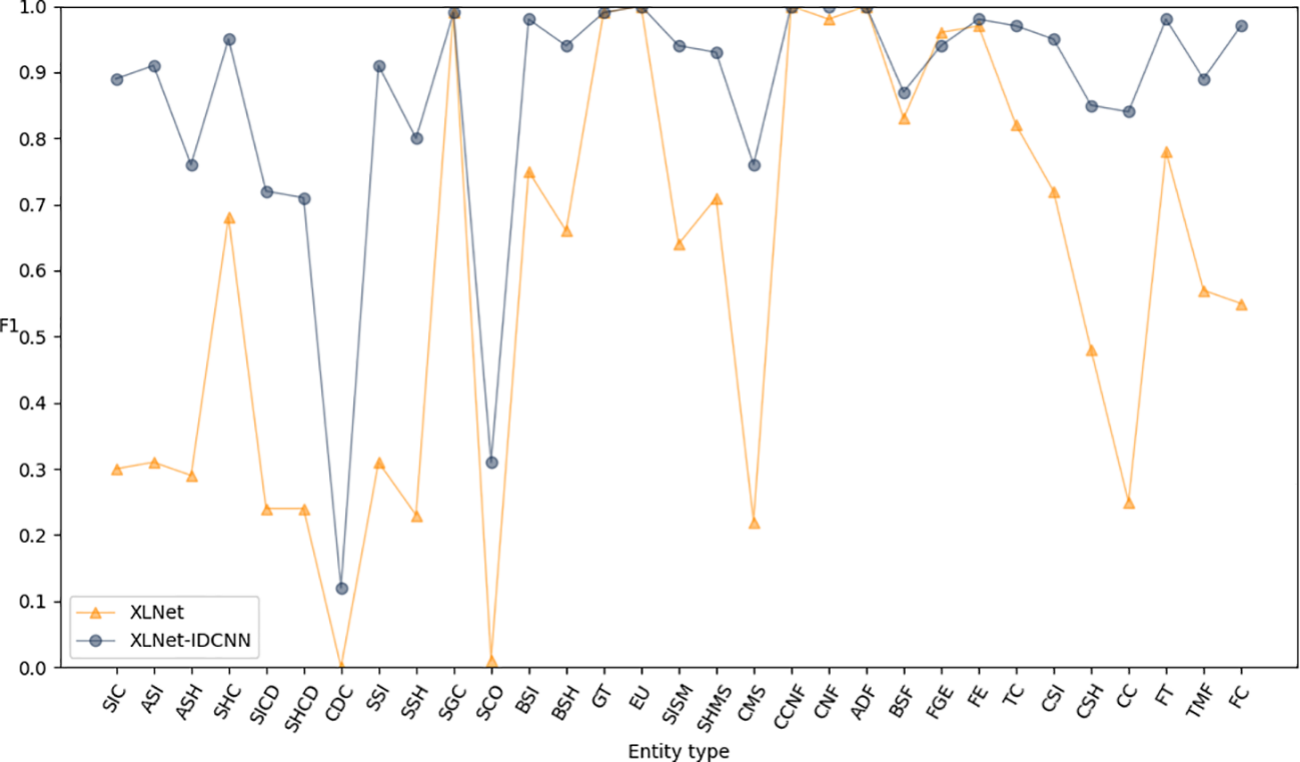
F1 values of XLNet and XLNet-IDCNN on different entities.

**Figures 3**–**5** present comparisons of P, R, and F1, respectively, between XLNet and XLNet-IDCNN in terms of entity recognition for different entity names. The results reveal that XLNet-IDCNN outperforms XLNet in terms of P, R, and F1 for the majority of entities. Specific analysis of the identification of each type of entity found that after adding the IDCNN layer, the following 28 types of entities were identified: the size of the ascus, ascospore size, ascospore shape, the shape of the ascus, size of the cystic disc, shape of the cystic disc, color of the cystic disc, sub-entity size, sub-entity shape, substrate growth cycle, sub-entity color, basidiospore size, basidiospore shape, size of mushroom stipe, shape of mushroom stipe, color of mushroom stipe, Chinese common name of the fungus, Chinese name of fungus, basic substance of fungus, fungal growth environment, fungus ecology, thickness of cap, cap size, cap color, cap shape, flesh thickness, texture of mushroom flesh, and flesh color. The P, R, and F1-scores of these 28 types of entities showed a significant improvement compared to XLNet. This improvement was especially seen for those entities with varying lengths and constructed as “value + unit” or “value + symbol + value + unit”.

This performance improvement is mainly attributed to the introduction of the IDCNN layer, which combines the ideas of Inception architecture and inflated convolution to give the model more powerful feature extraction and recognition capabilities: the Inception architecture captures multiple scales of input data by processing the input data in parallel with different scales of convolution kernels, while the inflated convolution enables the model to capture multiple scales of features by increasing the number of convolution kernels. kernel’s receptive field, enabling the model to better capture long-term dependencies in the input sequence.

The IDCNN layer shows its unique advantages for the entities mentioned above that have different lengths and special ways of word construction. On the one hand, due to the effect of the expansion convolution, the model is able to better capture the contextual information of these entities in the sequence, which improves the accuracy of recognition; on the other hand, the parallel processing mechanism of the Inception architecture enables the model to simultaneously take into account multiple scale features, which further strengthens the generalization ability and robustness of the model.

As a result, by introducing the IDCNN layer, our model demonstrates higher precision and recall in recognizing these entities with special word formation styles and length variations, thus achieving an overall performance improvement. This improvement not only enhances the recognition capability of the model, but also provides strong support for subsequent related research and applications.

#### 3.1.2 Performance comparison between XLNet-IDCNN and XIC

To further enhance the model’s performance and achieve globally optimal labeled sequences, a CRF was added on top of the IDCNN layer. To assess the impact of different epochs on model performance, experiments were conducted with 10, 20, 30, 40, 50, and 60 epochs, as detailed in **Table 4**. The results indicate that the model achieves optimal recognition performance with 50 epochs. Hence, the epoch parameter was set to 50 in all subsequent simulations. After 50 epochs, the P, R, and F1-scores reached 0.971, 0.986, and 0.979, respectively (**Table 5**). These results represent a significant improvement over XLNet-IDCNN (by 0.093, 0.025, and 0.062, respectively).

**Table 4:** Impact of different epochs on model performance.

Model	Epoch	P	R	F1
XIC	10	0.833	0.910	0.870
	20	0.893	0.949	0.920
	30	0.944	0.960	0.952
	40	0.960	0.980	0.970
	**50**	**0.971**	**0.986**	**0.979**
	60	0.969	0.982	0.976

The bold value represents the optimal result of the operation of this model.

**Table 5:** Adding conditions follows the results of airport named entity recognition.

Model	P	R	F1
XLNet-IDCNN	0.878	0.961	0.917
XIC	0.971	0.986	0.979

**Figure 6:**
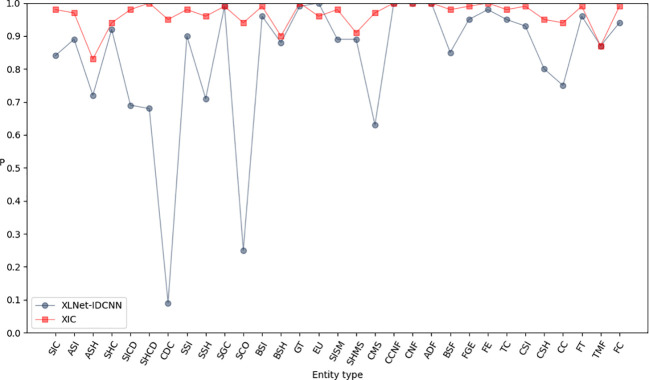
P-values of XLNet-IDCNN and XIC on different entities.

**Figure 7:**
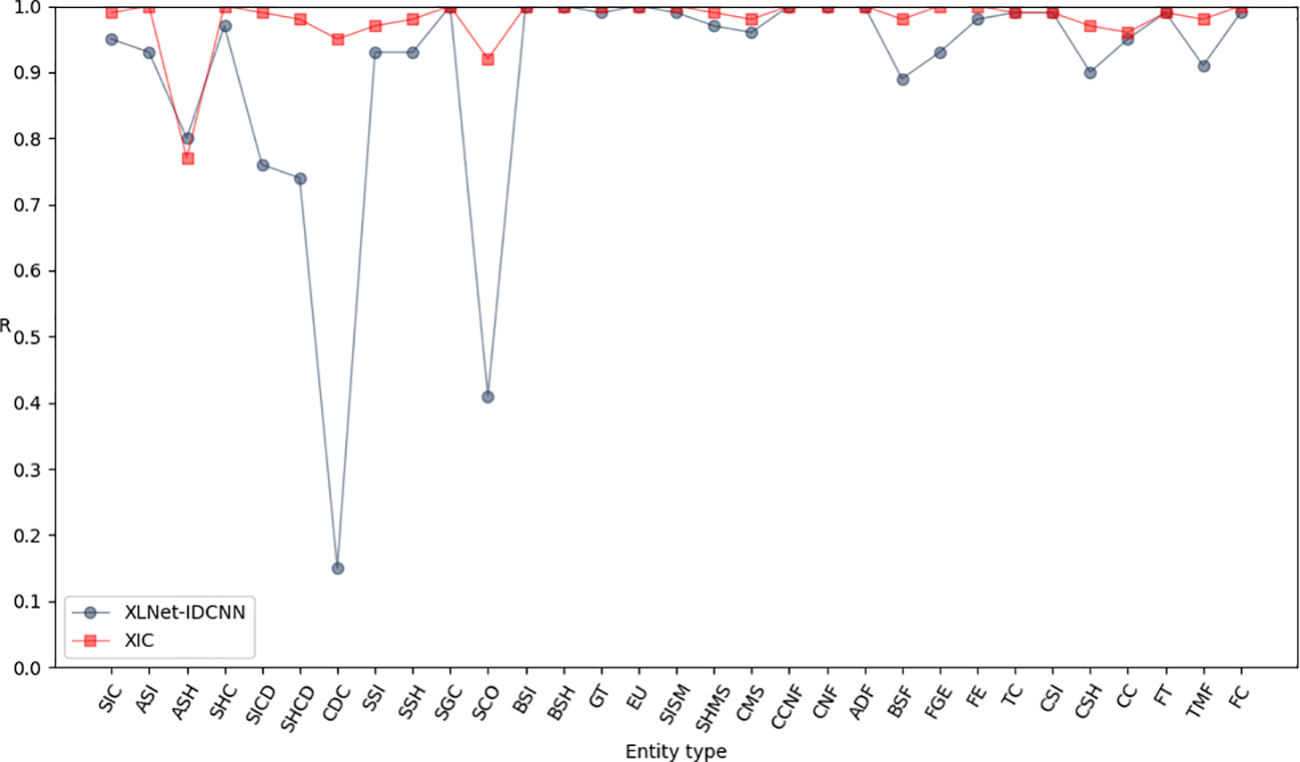
R-values of XLNet-IDCNN and XIC on different entities.

**Figure 8:**
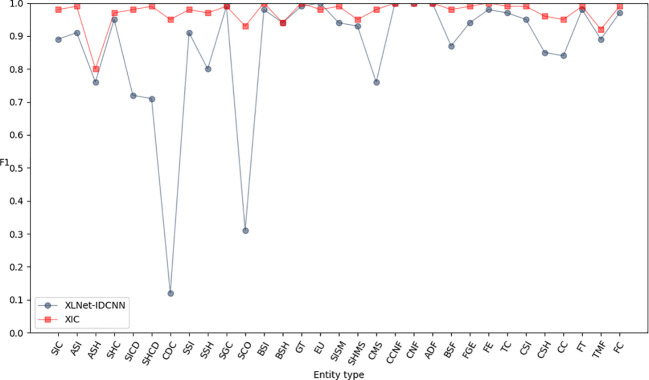
F1 values of XLNet-IDCNN and XIC on different entities.

**Figures 6**–**8** compare the P, R, and F1-scores, respectively, between XLNet-IDCNN and XIC for entity recognition with different entity names. The results highlight that the XIC model achieves higher P, R, and F1-scores than the XLNet-IDCNN model for most entities. Specific analysis of the recognition of each class of entities revealed that the addition of the CRF layer resulted in the inclusion of the size of the ascus, ascospore size, ascospore shape, the shape of the ascus, size of the cystic disc, shape of the cystic disc, color of the cystic disc, sub-entity size, sub-entity shape, growing time, size of mushroom stipe, shape of mushroom stipe, color of mushroom stipe, basic substance of fungus, fungal growth environment, fungus ecology, thickness of cap, cap size, cap color, cap shape, flesh thickness, and flesh color. There is a significant improvement in P for these 22 types of entities compared to XLNet-IDCNN. Among them, this enhancement is mainly attributed to the unique advantage of the CRF layer in the sequence annotation task.

The role of the CRF layer, as a key component in the sequence annotation task, is to combine contextual information to predict labels for each position in the sequence and ensure that the final predicted label sequence is globally optimal. When dealing with consecutive and interrelated entities such as cyst size and cyst spore shape, the CRF layer is able to capture the dependencies between these entities, thus enhancing the correctness of the prediction.

Specifically, the CRF layer enables the model to predict a label by considering the transfer probability between labels, which makes the model depend not only on the input features at the current position but also on the labels at the previous position. This feature enables the model to better handle the label dependency problem in sequence annotation tasks, thus avoiding label inconsistencies or errors that may occur in models such as XLNet-IDCNN.

As a result, by incorporating the CRF layer, our model not only improves the prediction accuracy of individual labels when recognizing the aforementioned 22 types of entities, but also ensures the coherence and consistency of the entire label sequence. This improvement makes our model show more superior performance in sequence labeling tasks, which provides strong support for research and applications in related fields.

### 3.2 Performance comparison of different models

To validate the superior performance of the XIC model proposed in this paper in recognizing named entities in edible fungus information text, several common NER models were compared. The models included in the comparison are BERT-CRF, BiLSTM-CRF, IDCNN-CRF, BERT-BiLSTM-CRF, and BERT-IDCNN-CRF. The experimental results are summarized in **Table 6**.

**Table 6:** Recognition results of different model named entities.

Model	P	R	F1
Bert-CRF	0.847	0.757	0.796
BILSTM-CRF	0.837	0.916	0.866
IDCNN-CRF	0.822	0.863	0.838
Bert-BILSTM-CRF	0.847	0.937	0.878
Bert-IDCNN-CRF	0.865	0.880	0.870
XIC	**0.971**	**0.986**	**0.979**

The bold value represents the optimal result of the operation of this model.

Although BERT-CRF and BiLSTM-CRF give better identification results for entities named in the edible bacterial information text, they only acquire short-range contextual characteristics, with a lack of long-distance and even global characteristic extraction. These models exhibit P scores of 0.847 and 0.837, R values of 0.757 and 0.916, and F1-scores of 0.796 and 0.866, respectively. The IDCNN-CRF model uses the traditional Word2Vec method to obtain word vectors. Nevertheless, the word vectors generated by this model are static, limiting feature extraction to the sentence level without considering internal features. Consequently, IDCNN-CRF exhibits the poorest performance in these comparative experiments, with P, R, and F1-scores of 0.822, 0.863, and 0.838, respectively. As an extension of BERT, XLNet effectively addresses limitations relating to the fine-tuning and pre-training processes. This not only strengthens the model’s capacity to capture long-distance dependencies, but also enhances the overall semantic representation. Hence, XIC surpasses the performance of BERT-IDCNN-CRF by 0.106, 0.106, and 0.109 in terms of P, R, and F1-scores, respectively. This notable performance improvement underscores the effectiveness of XLNet in recognition tasks. Integrating the insights discussed in Sections 3.2 and 3.3, the model presented in this paper not only addresses the challenge of inadequate feature capture between statements, but also circumvents labeling logic errors. Through the inclusion of the CRF layer, the model achieves globally optimal labels, resulting in more precise NER for texts related to edible fungus information.

### 3.3 Generalizability experiment

In order to verify the generality and robustness of the XIC model proposed in this paper, we compare and analyze the experimental results of the XLNet model, the XLNet-IDCNN model, and the XIC model on the public dataset MARS.

First, we recognize that the lack of Chinese datasets applicable to NER within the fungal domain is a challenge. This not only limits the direct application of the model, but may also negatively affect the generalization ability of the model. However, the MARS dataset we chose, although not directly targeting the edible fungi domain, provides us with a benchmark to evaluate the model performance as a generalized dataset containing multiple text types and named entities.

The results of the experiments on the MARS dataset are shown in **Table 7**, which show that the XLNet-IDCNN-CRF model achieves significant performance. Specifically, the model achieves high scores on key metrics such as F1-scores, P and R. These results indicate that even though the model was trained and tested on a non-domain-specific dataset, it is still able to accurately recognize named entities in text, which reflects the good generalization of the model.

**Table 7:** Experimental results of the model on the MARS dataset.

Model	P	R	F1
XLNet	0.622	0.815	0.706
XLNet-IDCNN	0.834	0.921	0.875
XIC	**0.856**	**0.937**	**0.894**

The bold value represents the optimal result of the operation of this model.

Further analysis shows that the pre-training mechanism of the XLNet model enables it to capture rich contextual information, which is crucial for improving the performance of the NER task. The IDCNN model, on the other hand, effectively captures local features in the text through its deep convolutional structure. Finally, the CRF layer, a commonly used structure in sequence labeling tasks, provides the model with globally optimal labeled sequences, which further improves the performance of the model.

In addition, we note that the model’s performance on the MARS dataset fluctuates less, which reflects the robustness of the model. Even when facing the challenges of different text types and named entities, the model is still able to maintain a stable performance.

## 4 Discussion

In the study of information related to edible mushrooms, it is crucial to accurately and efficiently recognize key entity information in text. To address this challenge, we propose the XIC model, which not only extends the boundaries of the XLNet architecture, but also significantly improves the model’s performance in the NER task of edible mushroom information by incorporating the IDCNN layer and the CRF layer.

The design of the XIC model takes into full consideration the characteristics of edible mushroom information text, especially the complex vocabulary and sentence structure it uses in describing entities. During the model construction process, we first chose XLNet as the infrastructure because it has demonstrated strong performance in natural language processing tasks. However, in order to further improve the effectiveness of the model in the edibles information NER task, we decided to introduce IDCNN layer and CRF layer on top of XLNet.

The IDCNN layer is unique in its ability to capture a wider range of dependencies by expanding the receptive field of the convolutional kernel. In edibles information text, the relationships between entities often span multiple words and sentences, which requires the model to have a strong ability to capture long-distance dependencies. The IDCNN layer is introduced to fulfill this need. By increasing the sensory field of the model, the IDCNN layer is able to capture more contextual information, which enriches the semantic information and improves the recognition accuracy of the model.

In addition, the IDCNN layer can also enhance the feature extraction capability of the model. In edibles information text, entities often consist of multiple words, and there may be complex dependencies between these words. The IDCNN layer is able to recognize entities more accurately by processing features at different scales in parallel and taking into account the interactions between multiple features at the same time.

The CRF layer, plays a crucial role in the NER task. Its main function is to predict the most probable labeling sequence given the input sequence. In the edible fungus information NER task, traditional prediction methods based on individual labels often have difficulty in obtaining optimal results due to the complexity and variety of relationships between entities. The CRF layer, on the other hand, is able to take into account the dependencies between labels, thus ensuring that the predicted label sequences are globally optimal.

In addition, the CRF layer is able to prevent labeling logic errors. In the NER task, labeling errors often lead to biased prediction results for the whole sequence. The CRF layer, however, by considering the transfer probability between labels, can ensure that the predicted labeled sequence is logically correct, thus improving the robustness and accuracy of the model.

The XIC model shows good interpretability in the prediction process. First, due to the introduction of the IDCNN layer, the model is able to capture more contextual information and give more accurate prediction results based on this information. This utilization of contextual information not only improves the recognition accuracy of the model, but also makes the prediction results more explanatory. Secondly, the addition of the CRF layer enables the model to consider the global optimality when predicting label sequences, thus avoiding the appearance of labeling logic errors. This global consideration makes the prediction results more stable and reliable, and also increases the interpretability of the prediction results.

In summary, the XIC model shows excellent performance in the edible mushroom information NER task. Its unique model architecture and efficient feature extraction capability enable the model to accurately identify the key entity information in the text. Meanwhile, the model also shows good interpretability in the prediction process, which provides strong support for us to understand and trust the prediction results of the model.

## 5 Conclusions

The practical contributions of this study to the research, cultivation, and popularization of knowledge related to edible fungus can be summarized as follows. First, we developed a method for extracting important entities from textual information related to edible fungus. This method was shown to improve the extraction efficiency compared with traditional approaches when dealing with large amounts of non-standardized data. This efficiency was crucial for processing and extracting valuable information from diverse and extensive datasets. Second, the proposed approach lays the foundation for constructing a knowledge graph specific to edible fungus. By identifying and extracting entities from textual information, this study has contributed to the creation of a structured representation of knowledge, enabling more organized and systematic information about edible fungus to be obtained. Third, the entities identified through NER were represented in the form of a knowledge graph. Knowledge graphs provide an intuitive and visual representation of the data model and structure. This representation can enhance the understanding of relationships and connections between different entities related to edible fungus. Finally, the authors intend to extend the NER method detailed in this paper to include relationship extraction in the context of information related to edible fungus. The ultimate goal is to build an intelligent Q&A system that leverages the extracted entities and their relationships, providing valuable insights and information in a user-friendly manner. In summary, the practical contributions of this study go beyond the development of the NER model, laying the groundwork for creating structured knowledge representations such as knowledge graphs, and envisioning the application of the proposed method in building intelligent systems for answering queries related to edible fungus. These contributions have the potential to significantly impact the accessibility and usability of knowledge in the field.

## Data availability statement

The original contributions presented in the study are included in the article/supplementary material, further inquiries can be directed to the corresponding author/s.

## Author contributions

HY: Data curation, Supervision, Writing – original draft, Writing – review & editing. CW: Investigation, Writing – original draft, Writing – review & editing. MX: Conceptualization, Supervision, Validation, Writing – original draft, Writing – review & editing.
